# Meetings of Societies

**Published:** 1905-06

**Authors:** 


### MEETINGS OF SOCIETIES.

### Bristol Medico-Chirurgical Society.

*March 8th, 1905.*

Dr. REGINALD EAGER, President, in the Chair.

Dr. H. WALDO showed a case of **leukæmia.** The patient was a woman of twenty-nine years of age. The disease became obvious soon after confinement. The spleen was very large. The leukæmia was of the spleno-medullary type, and may have started during pregnancy. The patient was not anæmic.—Dr. E. H. GROVES alluded to a case reported in the Obstetrical Society of London, in which an enlarged spleen, mistaken for a pelvic tumour, had been removed per vaginam.—Dr. MICHELL CLARKE thought the cases mentioned by the last speaker were probably examples of spleno-megaly, and had nothing to do with leukæmia.—Dr. JAMES SWAIN said the case shown had been under his care, the tumour having been suspected to be ovarian, but he had found it to be an enlarged spleen. If such an enlarged spleen gave rise to grave symptoms it might be advisable to remove it.

Dr. E. HEY GROVES showed a case of **multiple congenital amputations.** Though he had used the commonly accepted term of congenital amputation, he did not believe such cases really were amputations, they were rather due to imperfect development.

Dr. A. Ogilvy showed an **orbital exostosis** which had been removed from a patient shown at the last meeting of the Society.

Dr. James Swain showed a large **broad ligament fibroid.** The tumour weighed 1¼ lb.

Mr. J. Lacy Firth showed a **fibroma** which had been removed **from the abdominal wall.** The tumour was ovoid, encapsuled, and very dense. It had occupied the left iliac region, with its lower border close to Poupart's ligament, and was of the size of the closed fist. The external oblique aponeurosis had lain over it, the sub-peritoneal fat beneath and the arched fibres of the internal oblique and transversalis had been blended with it at its upper border.—Dr. Groves asked as to the relationship of the round ligament to the tumour, and Dr. Swain as to the duration of the symptoms.—Mr. Firth replied that he had not seen the round ligament as distinct from the tumour, and had not exposed the external abdominal ring. He had considered the possibility of the tumour having arisen in the round ligament. In any case the tumour was akin to those which had been described as fibromata of the abdominal wall. The tumour had been discovered by the patient four months before its removal, but it probably had existed a good deal longer than that.

Dr. H. Waldo read a paper on the **causes and treatment of baldness.** (For this paper see page 107.)—Dr. Kenneth Wills remarked that in considering the relationship between baldness and seborrhœa it was necessary to inquire whether the germs present were the cause or the result of the abundant secretion. Another question raised was whether or not the activity of the oil glands and that of the production of hair were alternatives. The seborrhœa of an infant protected the skin from the fluids in contact with it *in utero* and from external conditions after birth. Later the oil glands in part die away and hair begins to grow, and it was notable that infants with much oiliness of the skin remained almost bald for many months, and that as they became less oily the hair grew more abundantly, or they might retain the tendency to excessive oil production, and have repeated attacks of seborrhœic irritation and inflammation. At puberty the sebaceous glands frequently made themselves apparent by over-secretion, and acne in its various forms was the outcome. The sebaceous area of the face is the part where no hair grows. The negro is an oily man, but not hairy. In women at the menopause glandular activity is decreased, and the tendency to the growth of hair increases. From these considerations there appear to be certain grounds for supposing that the hair and oil production of the skin are alternatives. The fact that the sebaceous gland and the hair papilla are supplied with nourishment from the same or contiguous bloodvessels leads us to suppose that where the gland is excessively active there is less nourishment for the hair, and where the gland is dormant there is more chance for the hair to grow.

Dr. J. O. SYMES read a paper on **cytodiagnosis.** It had been claimed that from an examination of the cells present in pathological body-fluids it is possible not only to determine the nature of the morbid process, but also to forecast in some degree the subsequent progress of the disease. The speaker gave the results of his experience with this method of examination. The fluids examined were centrifugalised and the sediment spread in films on glass slides and the films stained. The distinctions between the various forms of pleural effusion as revealed by cytodiagnosis were described. In tubercular effusions the lymphocytes predominate; in effusions due to other micro-organisms there is a high percentage of polymorphonuclear leucocytes; in mechanical transudations large numbers of endothelial cells are found; and in cancer of the pleura certain cells characteristic of the new growth are to be detected. In a similar manner the speaker gave his results in the examination of ascitic fluid, urine, hydrocele fluid, and cerebro-spinal fluid.

*April 12th, 1905.*

Dr. REGINALD EAGER, President, in the Chair.

Dr. E. C. WILLIAMS showed a case of **adiposis dolorosa.**

Dr. E. H. STACK showed for Dr. P. WATSON WILLIAMS a case of **myxœdema** in a child aged five years.

Dr. W. H. C. NEWNHAM showed two **specimens of malignant disease of the uterus** removed by operation. One of these had been obtained by hysterectomy from a woman of 52. She had been sent to the speaker for uterine hemorrhage. Examination of the specimen showed the presence of a round-celled sarcoma in the upper zone of the uterus. Seven months later the patient was well, and there were no signs of recurrence. The other specimen had been removed from a single woman, and showed a carcinoma arising in a fibroid of the uterus. The patient had been seen ten years previously, and then had an ordinary fibroid. During the operation, on March 14th, 1905, the patient had been transfused. She was still living. Such a case suggested the question, Ought we not to remove all fibroids of the uterus? —Mr. MORTON did not think the view could be accepted that all fibroids, even though only of moderate size, required removal. As the previous speaker had remarked, no one could promise a patient that all her troubles would cease at the menopause; but in many cases a fibroid gave no trouble, or if it did give trouble, that often ceased at the menopause.

Dr. JAMES SWAIN showed a **specimen of sarcoma of the kidney** removed by the anterior incision. The specimen weighed about 1½ lb., and microscopically was found to be a round-celled sarcoma without the evident presence of any of the striped muscular tissue which is not infrequently found in such specimens. There was a considerable amount of shock at the time of the operation, but the child rapidly recovered. The speaker remarked that it was not long since such an operation was considered unjustifiable, but it is desirable to operate in all cases where the general condition is fairly good and the rapidly-enlarging tumour has not been long in existence. —Mr. Morton referred to three cases of renal sarcoma in which he had operated. One was a case of nephrectomy in a child eighteen months of age, which was published in the *British Medical Journal*, 1900, vol. i., p. 249. The tumour was the size of a cocoanut, and the top of the kidney projected from it. It was an adeno-sarcoma. There was very little shock from the operation, although the child was so young. Death occurred five months later from tuberculosis, and no recurrence of the growth was found *post mortem*. The other case of nephrectomy for sarcoma was at the other extreme of life—a woman 68 years of age. This tumour was also removed by the anterior abdominal incision. She made a good recovery from the operation, but died three months later without evidence of recurrence; but there was no *post mortem*. The third case was of interest in connection with its diagnosis. A child, aged 3 years, had a large lumbar swelling, which Mr. Morton thought was sarcoma, and he proceeded to explore it by the anterior abdominal incision. It then felt cystic; he put an exploring syringe into it from the loin, and drew off a syringeful of fluid like clear tea. Thinking it was a hydronephrosis, he explored it from the loin, but found a degenerated hemorrhagic cyst in a sarcomatous kidney. It was not a suitable case for nephrectomy.

Mr. Paul Bush showed a **specimen of soft fibroid of the uterus**, weighing 15 lb. when removed, from a lady just under 80 years of age. She had had a uterine tumour for thirty or forty years. Hysterectomy had been performed and the patient had made an excellent recovery. The operation was performed on account of considerable increase in size of the tumour, associated with bladder and rectal symptoms and signs of severe pressure on nerves.

Dr. J. Michell Clarke read a paper on **Certain symptoms of tumour of the cerebellum with illustrative cases.** (For this paper see p. 97.)

Dr. W. Kenneth Wills read a paper on **the treatment of acne vulgaris.** (For this paper see p. 113.)

Dr. Bertram M. H. Rogers read notes on two cases of **Congenital heart disease** and showed the specimens. In the first case there was complete atresia of the tricuspid orifice, patency of the foramen ovale and a perforation at the "undefended space" between the ventricles. The right ventricle was rudimentary, the other cavities distended and hypertrophied, especially the right auricle and left ventricle. The ductus arteriosus was closed. The heart weighed 3½ ounces. The second specimen showed practically complete atresia of the pulmonary orifice, patency of the foramen ovale, and a large perforation at the "undefended space" between the ventricles. The aorta arose from both ventricles. Both ventricular walls were much hypertrophied, especially the right. The valves appeared normal. The ductus arteriosus was closed. The pulmonary artery and its main branches were filled with old organised clot. The pulmonary veins were small. The heart weighed 4½ ounces.

*May 10th, 1905.*

Dr. Reginald Eager, President, in the Chair.

The meeting was devoted to the exhibition and discussion of cases.

Dr. J. O. Symes showed cases of **acute rheumatoid arthritis.**

Dr. Carey Coombs showed an infant with an **abdominal tumour.**

Dr. F. H. Edgeworth showed cases of (*a*) **head-nodding and nystagmus,** and (*b*) a **cretin undergoing treatment with thyroid extract.**

Mr. G. Munro Smith showed a case of **cerebritis.**

Dr. J. J. S. Lucas showed cases of (*a*) **macroglossia** and (*b*) **unexplained œdema of the legs.**

Dr. Kenneth Wills showed cases of (*a*) **linear nævi,** (*b*) **lupus cured by X-rays,** (*c*) **adenoma sebaceum treated by high frequency currents,** (*d*) **symmetrical dystrophy of nails,** (*e*) **rodent ulcer treated by X-rays.**

Dr. H. Waldo showed cases of (*a*) **tuberculosis cutis (generalised),** (*b*) **tinea circinata,** (*c*) **lupus vulgaris,** (*d*) **cheiro-pompholyx.**

Mr. F. R. Cross showed four cases of **orbital tumour.**

Mr. J. Paul Bush showed cases of (*a*) **post-pharyngeal abscess** treated by external incision, and (*b*) **pyonephrosis** of eleven years' standing.

Dr. J. A. Nixon showed a case of **multiple nævi with pigmentation.**

Dr. J. Michell Clarke showed a case of **lesion of the fifth cervical nerve root.**

Mr. R. G. P. Lansdown showed cases of (*a*) **excision of the rectum,** and (*b*) **birth-fracture of the skull** treated by Nicoll's method.

Mr. H. F. Mole showed a case probably of **mycosis fungoides.**

Mr. C. H. Walker showed a case of **orbital tumour.**

Mr. J. Lacy Firth showed cases of (*a*) **epispadias** cured by a series of operations, and (*b*) **double frontal sinusitis and left maxillary empyema** treated by operation.

J. Lacy Firth.
H. F. Mole (*Hon. Sec.*).

